# Evaluating the Utility of a Psychoeducational Serious Game (SPARX) in Protecting Inuit Youth From Depression: Pilot Randomized Controlled Trial

**DOI:** 10.2196/38493

**Published:** 2023-03-09

**Authors:** Yvonne Bohr, Leah Litwin, Jeffrey Ryan Hankey, Hugh McCague, Chelsea Singoorie, Mathijs F G Lucassen, Matthew Shepherd, Jenna Barnhardt

**Affiliations:** 1 LaMarsh Centre for Child and Youth Research York University Toronto, ON Canada; 2 Department of Psychology Faculty of Health York University Toronto, ON Canada; 3 Institute for Social Research York University Toronto, ON Canada; 4 Nunabox Iqaluit, NU Canada; 5 Faculty of Wellbeing, Education and Language Studies The Open University Milton Keynes United Kingdom; 6 School of Psychology Massey University Auckland New Zealand

**Keywords:** psychoeducation, cognitive behavioral therapy, Inuit youth, Nunavut, depression, suicide, resilience, serious game, youth, mental health, teen, adolescent, pilot study, community

## Abstract

**Background:**

Inuit youth in Northern Canada show considerable resilience in the face of extreme adversities. However, they also experience significant mental health needs and some of the highest adolescent suicide rates in the world. Disproportionate rates of truancy, depression, and suicide among Inuit adolescents have captured the attention of all levels of government and the country. Inuit communities have expressed an urgent imperative to create, or adapt, and then evaluate prevention and intervention tools for mental health. These tools should build upon existing strengths, be culturally appropriate for Inuit communities, and be accessible and sustainable in Northern contexts, where mental health resources are often scarce.

**Objective:**

This pilot study assesses the utility, for Inuit youth in Canada, of a psychoeducational e-intervention designed to teach cognitive behavioral therapy strategies and techniques. This serious game, SPARX, had previously demonstrated effectiveness in addressing depression with Māori youth in New Zealand.

**Methods:**

The Nunavut Territorial Department of Health sponsored this study, and a team of Nunavut-based community mental health staff facilitated youth’s participation in an entirely remotely administered pilot trial using a modified randomized control approach with 24 youths aged 13-18 across 11 communities in Nunavut. These youth had been identified by the community facilitators as exhibiting low mood, negative affect, depressive presentations, or significant levels of stress. Entire communities, instead of individual youth, were randomly assigned to an intervention group or a waitlist control group.

**Results:**

Mixed models (multilevel regression) revealed that participating youth felt less hopeless (*P*=.02) and engaged in less self-blame (*P*=.03), rumination (*P*=.04), and catastrophizing (*P*=.03) following the SPARX intervention. However, participants did not show a decrease in depressive symptoms or an increase in formal resilience indicators.

**Conclusions:**

Preliminary results suggest that SPARX may be a good first step for supporting Inuit youth with skill development to regulate their emotions, challenge maladaptive thoughts, and provide behavioral management techniques such as deep breathing. However, it will be imperative to work with youth and communities to design, develop, and test an Inuit version of the SPARX program, tailored to fit the interests of Inuit youth and Elders in Canada and to increase engagement and effectiveness of the program.

**Trial Registration:**

ClinicalTrials.gov NCT05702086; https://www.clinicaltrials.gov/ct2/show/NCT05702086

## Introduction

Inuit are Indigenous Peoples of the Arctic and subarctic regions of Greenland, Alaska, and Canada. In Canada, they are recognized as a distinctive group of Indigenous Canadians—alongside First Nations and Métis—primarily occupying Labrador, Northern Quebec, the Northwest Territories, and Nunavut. Pervasive youth suicide is the most urgent challenge facing Inuit in Canada today, a public health emergency of epidemic proportions [1,2]. Death by suicide in Inuit communities has spiked over the last century [3,4], currently at 9 times the national average and with male youth aged 15-24 being the most affected [5]. Under the fallout of colonialism, Inuit youth in Canada face more mental health challenges than non-Indigenous populations, due to cross-generational impacts of residential schools and multiple continuing systemic inequities [6,7]. The government era of the 1950-60s and beyond, during which the Canadian government forced Inuit into aggregated settlements, imposed a welfare state, and separated Inuit children from their families, has had lasting effects on relationships within family and community, strained kinship across generations and between genders, and left generational gaps in mental health–supporting communications between youth and their parents and Elders [3]. Further, Indigenous youth in general tend to be among those most reluctant to seek help when experiencing distress [8]. This lack of help-seeking may be due in part to a struggle to recognize symptoms of depression, which may present as anger in Inuit youth [4,9].

Those living in remote Northern communities often go without access to the mental health supports available elsewhere in Canada [10] for a number of reasons. First, few health care professionals train or practice in the Canadian North, and frontline workers often experience “burnout” due to excessive workload and personnel shortages [11]. As a result, youth in Nunavut have limited access to specialized services and are often sent out of their communities when they experience severe mental health symptoms [10,12-14]. Finally, adolescents in small, remote communities are especially prone to avoiding professional health services, as they may fear compromised confidentiality due to a lack of privacy and the stigma associated with mental illness. One study found that of all youth who committed suicide in Nunavut in the past decade, only 12% had received medication and 17% had been hospitalized in relation to their mental health, while 89% had not received any mental health support at all [14,15]. Given the reality that there are relatively few frontline mental health workers available in Nunavut communities and stigma impeding access to existing services, it is imperative to identify and develop preventive interventions for depression that are not only effective and culturally appropriate, but also easily accessible [11].

**Figure 1:**
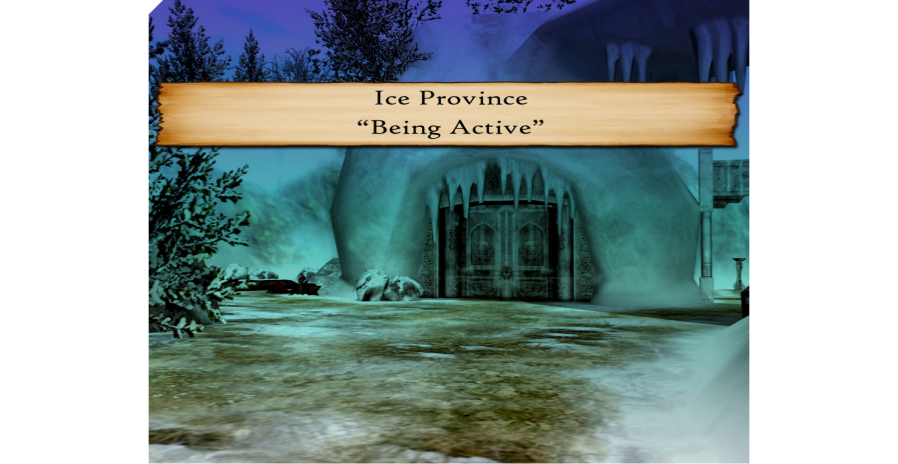
Screenshot of a SPARX CBT module. Used with permission of the copyright owner © Auckland UniServices Limited.

**Figure 2:**
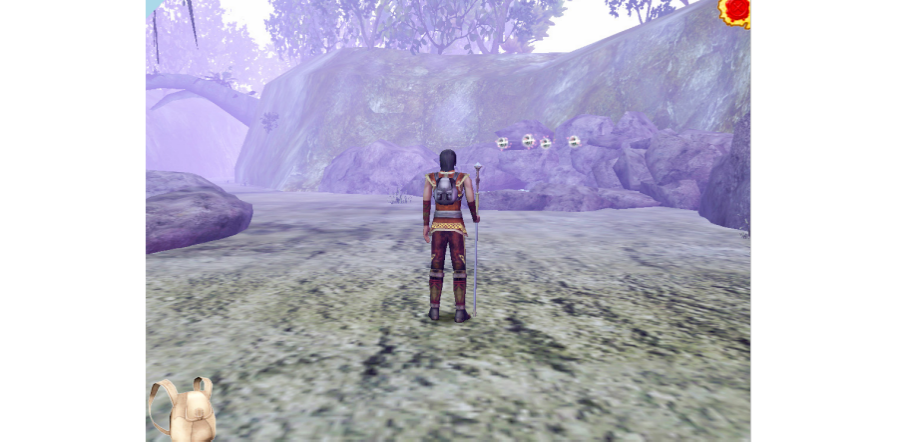
Screenshot of approaching GNATs. Used with permission of the copyright owner © Auckland UniServices Limited.

To address the dearth of available in-person mental health support options, this study aims to examine the usefulness of an e-intervention, SPARX [16,17], in Nunavut. SPARX (Smart, Positive, Active, Realistic, X-Factor thoughts) is a psychoeducational *serious game* (an e-intervention that utilizes gaming for serious purposes) [18] that teaches established cognitive behavioral therapy (CBT) strategies and techniques across 7 levels or modules (see Figure 1 for an example). The game is designed to address depressive symptoms in youth by helping them cope with negative thoughts and feelings, represented in the game as GNATs—gloomy negative automatic thoughts (Figure 2) [16]. SPARX was originally designed and developed at the University of Auckland with the specific needs of certain underserved groups of youth in mind, including Māori Rangatahi, the Indigenous young people of Aotearoa, New Zealand. SPARX’s development included a Māori co-creator (MS), input from Māori CBT experts, and cultural guidance from a *kaumatua* (ie, respected Elder). Moreover, the game development company was led by a Māori woman and the voice actor (virtual therapist/instructor) was a Māori; additionally, the Māori symbolism, presented in a fantasy format, was woven throughout the intervention [17]. With these very specific cultural foundations in mind, the goal of this pilot study was to evaluate the effectiveness of the original version of SPARX in boosting resilience against depression among Inuit youth in Canada in 11 Nunavut communities. We aimed to assess whether Inuit youth who completed the SPARX program experienced increased resilience and showed a decrease in risk factors related to depression, specifically cognitive distortion, emotional dysregulation, hopelessness, rumination, self-blame, other-blame, and catastrophizing [19].

The Nunavut Territorial Department of Health sponsored this study, and a team of Nunavut-based community mental health staff facilitated youth’s participation in an entirely remotely administered pilot trial. A total of 48 youths from 11 Nunavut communities completed an evaluation of the utility of SPARX in modifying dysfunctional cognitions, reducing symptoms of depression, and enhancing resilience. All youth completed conventional pre- and postintervention measures assessing their current mental health status. Measures were selected based on their use in earlier effectiveness studies [16,17]. Quantitative and qualitative data were collected and used to evaluate the program’s preliminary effectiveness based on the following hypotheses:

- Youth who completed the SPARX program were expected to experience a decrease in depressive symptoms as well as risk factors related to depression, as measured by the Centre for Epidemiologic Depression Scale-Revised (CESD-R), the Hopelessness Scale for Children (HSC), and the Cognitive Emotion Regulation Questionnaire-Short (CERQ-Short; see the “Measures” section).
- Youth who completed the SPARX program were expected to experience an increase in factors related to resilience, as measured by the shortened, 12-item version of the Child and Youth Resilience Measure (CYRM-12).

## Methods

### Participants

All 25 Nunavut communities were initially recruited to participate in this study; 11 communities (Baker Lake, Cambridge Bay, Grise Fiord, Hall Beach, Igloolik, Kugaaruk, Kugluktuk, Naujaat [Repulse Bay], Taloyoak, Qikiqtarjuaq, and Resolute) completed the pilot trial. The remaining 14 communities did not complete data collection because of an inability to recruit youth, because there was no local adult facilitator able to support youth, or because facilitators had to leave the project due to extraneous circumstances. Communities were chosen based on feasibility and required both an interested community facilitator with the time to commit to SPARX and youth who were interested in the project. As many as 3-4 youths were recruited in each community with a projected participant count of 40.

Youth participants were between the ages of 13 and 18 and had been identified by the community facilitators as exhibiting low mood, negative affect, depressive presentations, or significant levels of stress. Youth had to demonstrate sufficient English language comprehension to use and understand SPARX. Although such an approach is not reflective of Indigenized research generally, these inclusion criteria were suggested by local facilitators due to the heavy use of the English language within the SPARX game. Initially, youth were excluded if they showed limited cognitive abilities, psychotic presentation, severe depression, or elevated suicide risk, or if they were currently receiving or had previously received CBT, interpersonal therapy, or antidepressant medication within the past 3 months. However, due to safety and ethics concerns about vulnerable youth feeling left out, at the discretion of local mental health experts on the team, these exclusion criteria were relaxed. The 25 facilitators were appointed by staff at the Department of Mental Health, Government of Nunavut. In total, 24 of 48 youths who initially signed up completed the study (see the “Design” section for attrition rates at each time point).

### Procedure

A preproject planning meeting was held in Toronto with managers of the Territorial Department of Health, a team of community mental health staff from Nunavut, and potential on-site facilitators on March 27, 2014. Procedural aspects of the SPARX pilot project were based on contributions and feedback from attendees of this meeting. The meeting focused on the importance of the SPARX project in Nunavut communities, how the SPARX project could achieve cultural appropriateness, and what traditional and cultural knowledges must be incorporated to gain youth trust and participation. In a collaboration among Nunavut Department of Mental Health staff, community stakeholders, and the coordinating team at York University, the design described in this work was adopted at the planning meeting.

### Ethics Approval

Approval for the SPARX pilot was obtained through the Human Participants Sub-Committee of the York University Office of Research Ethics (York University HPRC Certificate No. 2015-070). Approval was also obtained from the university’s Advisory Group for Research Involving Indigenous Peoples. This subcommittee is guided by Chapter 9 of the Tri-Council Policy Statement “Research involving the First Nations, Inuit, and Métis Peoples of Canada,” and its own Guidelines for Research Involving Indigenous Peoples. In addition, the research team received approval in the form of a research license from the Nunavut Research Institute, which represents all 25 Nunavut communities (license number 02 004 15N-M).

### Design

A modified randomized control approach was adopted for this trial. Community facilitators, responsible for recruiting youth and facilitating gameplay, were trained prior to youth recruitment and played SPARX to familiarize themselves with the game. Once training was complete, community facilitators recruited youth, secured consent, and administered preintervention measures. Entire communities, instead of individual youth, were randomly assigned to an intervention group (group A) or a waitlist control group (group B), which is why a modification was made to the randomized control trial, as described in the following section.

**Figure 3:**
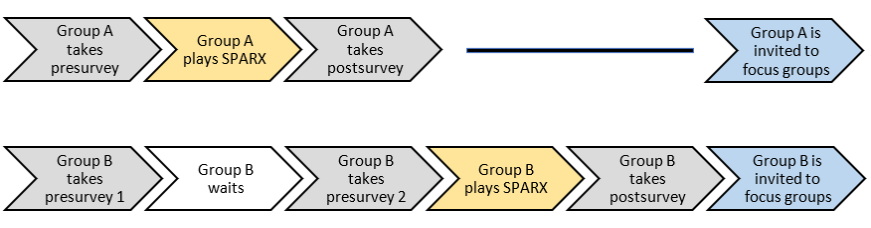
Sequence of SPARX play.

The sequence of SPARX play differed for the youth depending on whether they were in group A or group B (Figure 3). Both groups completed the preintervention surveys at time 1, the beginning of the study. Group A youth then began to play SPARX for 7 weeks while group B waited. During their wait time of 7 weeks, group B youth were not required to participate in any SPARX activities or meet with the community facilitator, and they were provided with no additional SPARX-related information. After 7 weeks (time 2), group A youth completed postintervention surveys, while group B youth began their engagement with SPARX by first completing an additional set of preintervention surveys, immediately followed by 7 weeks of SPARX play. Once group B youth completed their SPARX gameplay (time 3), they next completed postintervention surveys. Attrition rates were 35% (7/20) for group A (the intervention group) at time 2, 32% (9/28) for group B (the waitlist control group) at time 2, and a further 47% (9/19) for group B at time 3. This was due to high community facilitator turnover, timing of holidays, loss of interest, and arising community crises that took facilitators away from supporting SPARX usage. Similar attrition rates are not uncommon in pilot studies in remote communities.

Youth played SPARX on the laptops in either the community facilitator’s office or in an office in their school. Youth were asked to engage in SPARX play once a week; however, due to complications in some communities (eg, crises, snowstorms), some youth played more or less frequently than this. Youth engaging in SPARX were always supported and accompanied by a community facilitator, given the exploratory nature of this novel study. This assistance was provided to ensure that any support youth needed with gameplay or with their mental health difficulties was prudently available while this new intervention was being piloted. The sequence of SPARX play is detailed in the following sections.

### Measures

Pre- and postintervention surveys consisted of the same set of measures at both time points. Measures were either completed online via SurveyMonkey or in printed paper format. Community facilitators were on-site with the youth while they completed the measures to provide support such as reading questions out loud to the youth, or talking through answers if any youth felt vulnerable, at risk, or triggered by the content of the questions. Measures were selected based on their relevance; use in earlier, related studies; established psychometric properties for youth; sensitivity to change over time; ease of administration; length and clarity; and constraints based on funding sources.

The goal of this pilot study was to replicate, as best as possible in this new context, the studies conducted with Māori youth [16] to assess the potential usefulness of SPARX as an intervention with Inuit youth in Canada. It was clear to the researchers that should funding subsequently be obtained for a more comprehensive study, more culturally appropriate questionnaires would then be developed, together with Inuit communities, to assess youth depression and resilience [20]. As of the time of the writing of this paper, that has indeed happened, and a study of a culturally adapted version of SPARX for Inuit youth is underway [21]. In the absence of culturally appropriate outcome measures to assess mental health factors related to suicidality in Inuit youth, and to remain as consistent as possible with existing studies of SPARX conducted in New Zealand, the following primary outcome measures were chosen to assess changes in depression, hopelessness, resilience, and cognitive emotion regulation within these communities.

The CESD-R [22] is a succinct, 20-item, self-report scale that aims to measure current depressive symptomatology. Each item is a symptom associated with depression. The CESD-R has acceptable internal consistency (Cronbach *α*=.85) and test-retest reliability [23]. Validity was also established based on correlations with other assessment tools [24]. A sample item includes, “In the past week, I was bothered by things that don’t usually bother me.” Although it has not yet been evaluated within Inuit communities, the CESD-R is the most commonly used measure of depression among Indigenous youth, and has demonstrated a good model fit, with a root-mean-square error of approximation [25] of 0.6.

The HSC [26] contains 17 true-false items, which describe negative future expectations and negative present attitudes. Scores range from 0 to 17. Internal consistency is *α*=.97 and test-retest reliability is *r*=0.52. The HSC correlated positively with depression (*r*=0.58) and negatively with self-esteem measures (*r*=–0.61) and social skills (*r*=0.39) [26]. A sample item includes, “I might as well give up because I can’t make things better for myself.” This questionnaire has good internal consistency with other Indigenous youth (*α*=.86) but has not been evaluated with Inuit communities [27].

The CERQ-Short [19] is an 18-item measure used with adolescents and has good psychometric properties. It is composed of 9 coping styles, which are each coded as separate subscales of emotion regulation. These include Self-blame (score 1), Acceptance (score 2), Focus on Thought/Rumination (score 3), Positive Refocusing (score 4), Refocus on Planning (score 5), Positive Reappraisal (score 6), Putting Into Perspective (score 7), and Catastrophizing (score 8). Cronbach *α* coefficients range from .68 to .83 for the 9 subscales. Test-retest reliability ranged [19] from *r*=0.48 to 0.65. A sample item includes, “I keep thinking about how terrible it is what I have experienced.” The CERQ-Short has been used with selected Indigenous populations; however, no official reliability or validity values have been published and it has not been evaluated with Inuit communities.

The CYRM-12 [28] is a 12-item self-report measure that includes 3 dimensions (Individual, Relational, and Contextual) that reflect the major categories of resilience. Raters choose whether the sentences describe them and endorse either “No,” “Sometimes,” or “Yes.” The scale was developed for use with youth between ages 13 and 22 [28]. Internal consistency had Cronbach *α* ranging [29] from .65 to .91. A sample item includes, “When things don’t go my way, I can fix it without hurting myself or other people (for example hitting others or saying nasty things).” The CYRM-12 has frequently been used with Indigenous populations, showing good content and internal validity, but has not been evaluated with Inuit communities [28].

### Data Analysis

Because of the challenges encountered with youth recruitment, and the knowledge that an ideal sample size would likely be unattainable, no formal power calculations were conducted prior to beginning this study. To analyze mental health changes in Inuit youth in Canada over time, as measured through the 4 questionnaires, paired samples *t* tests (both 1- and 2-tailed—see below) comparing pre- and postintervention outcomes and repeated measures ANOVAs were conducted via SPSS Statistics version 26 (IBM Corp). ANOVAs allowed for examining the effect of time interacting with the intervention group or the waitlist control group. Paired samples *t* tests and ANOVAs pooled group A and group B data to account for missing data across groups and fewer completed postmeasures from group B youth. As a result of pooled data, there was some loss in accuracy due to the omission of select cases that did not fit the structure required by these simpler statistical methods.

Given the structure of the study—the comparison of the intervention group (group A) and the waitlist group (group B) at all (up to 3) time points for the pre- and postmeasures (group A: premeasure at time 1, postmeasure at time 2; group B: premeasure at time 1, premeasure at time 2, and postmeasure at time 3)—mixed models (multilevel regression) using pooled data were conducted via SPSS software. Multilevel regression models were appropriate given their ability to detect individual differences across different treatment options (time points for youth in group A vs group B). Results of the multilevel regression models are considered the most accurate as they use all the nonmissing data (important with a small sample size), reflect the full design of the study, and allow for comparison between group A and group B at different time points. Multilevel regression models were fit with (1) a randomly varying intercept for each youth to account for their differing initial levels of personally identified health and (2) an intervention indicator variable (0 for not having started playing the SPARX program vs 1 for completing the treatment of playing SPARX) to account for the effect of the treatment (ie, engaging in SPARX). A time variable was not needed as there were at most 2 (group A) to 3 (group B) measurement occasions with a horizontal, time-independent trajectory after accounting for the effect of the intervention. The assumptions of the mixed models were checked (eg, normality of the residuals) and found reasonable.

### Researcher Characteristics

Enduring colonial approaches to research in Canada have severely diminished Inuit sovereignty with respect to research activity in the Inuit homeland [30]. In the spirit of accountability and reconciliation, we believe it is important to acknowledge that the research team is diverse and consists of Indigenous and non-Indigenous team members. CS, our Inuit community liaison, is an Inuk and a longstanding research partner with YB and her team of researchers at York University. MS is a Māori researcher who co-developed the SPARX program with Indigenous youth in New Zealand. JB is an Indigenous status First Nations student, born and raised in Tyendinaga/Kenhteke Mohawk Territory. YB, LL, JRH, HM, and MFGL are White researchers from the South (*Qallunaat*).

## Results

### Overview

Table 1 provides a summary of the baseline and postintervention results for participants in groups A and B. Mean scores and the SDs for the 4 outcome measures (by subscale for the CERQ-Short) are provided below.

**Table 1:** Descriptive statistics by group and time point.

Outcome measure	Group A time 1 (n=20), mean (SD)	Group A time 2 (n=13), mean (SD)	Group B time 1 (n=28), mean (SD)	Group B time 2 (n=19), mean (SD)	Group B time 3 (n=10), mean (SD)
CYRM-12^a^: Resilience	44.70 (8.04)	41.07 (10.22)	43.32 (7.54)	43.62 (9.44)	45.30 (8.42)
HSC^b^: Hopelessness	4.52 (2.75)	3.79 (3.09)	5.39 (2.50)	4.73 (3.34)	3.67 (2.58)
CESD-R^c^: Depression	22.90 (8.30)	23.21 (8.86)	25.50 (8.54)	23.68 (8.58)	21.20 (7.60)
CERQ-Short^d^ subscale 1: Self-blame	4.90 (2.70)	5.00 (1.84)	6.04 (2.13)	6.10 (2.13)	4.20 (1.75)
CERQ-Short subscale 2: Acceptance	6.30 (2.24)	5.64 (2.20)	7.39 (2.33)	7.26 (2.37)	7.90 (1.73)
CERQ-Short subscale 3: Focus on Thought/Rumination	5.70 (2.39)	5.07 (1.90)	7.07 (2.16)	6.68 (2.29)	6.00 (1.89)
CERQ-Short subscale 4: Positive Refocusing	5.60 (1.79)	5.21 (2.19)	5.29 (1.90)	5.58 (2.27)	6.40 (2.07)
CERQ-Short subscale 5: Refocus on Planning	7.10 (2.40)	5.86 (2.51)	6.50 (2.23)	6.58 (2.24)	6.10 (2.42)
CERQ-Short subscale 6: Positive Reappraisal	7.40 (2.35)	7.07 (2.30)	8.00 (1.61)	7.68 (1.82)	6.70 (2.47)
CERQ-Short subscale 7: Putting Into Perspective	6.40 (2.39)	5.79 (2.01)	6.43 (2.28)	6.32 (1.60)	5.50 (2.37)
CERQ-Short subscale 8: Catastrophizing	5.50 (2.70)	4.29 (1.07)	6.50 (2.46)	5.89 (2.51)	5.60 (2.41)

^a^CYRM-12: Child and Youth Resilience Measure (12-item).

^b^HSC: Hopelessness Scale for Children.

^c^CESD-R: Centre for Epidemiologic Depression Scale-Revised.

^d^CERQ-Short: Cognitive Emotion Regulation Questionnaire—Short.

### Hypothesis One: Youth Who Completed the SPARX Program Were Expected to Experience a Decrease in Depressive Symptoms and Risk Factors Related to Depression, as Measured With the CESD-R, HSC, and CERQ-Short Questionnaires

Participating youth reported feeling less hopeless, and engaged in less self-blame, rumination, and catastrophizing following the SPARX intervention (Table 2). The intervention was a statistically significant predictor of the HSC score (*b*=–1.08; *t*_51.1_=–2.17; *P*=.02<.05; 1-tailed based on the *a priori* hypothesized direction of improvement in health). The intervention decreased the HSC score: Hopelessness by 1 (1.08) unit. The intervention was a statistically significant predictor of the CERQ-Short subscale score 1 (Self-blame; *b*=–0.86; *t*_53.2_=–1.95; *P*=.03<.05; 1-tailed). The intervention tended to decrease the CERQ-Short subscale score 1: Self-blame by close to 1 (0.86) unit. The intervention was a statistically significant predictor of the CERQ-Short subscale score 3 (Focus on Thought/Rumination; *b*=–0.77; *t*_53.6_=–1.84; *P*=.03<.05; 1-tailed). The intervention was a statistically significant predictor of the CERQ-Short subscale score 8 (Catastrophizing; *b*=–0.90; *t*_52.7_=–2.00; *P*=.02<.05; 1-tailed).

For scores on the CESD-R, and the CERQ-Short subscale scores 2, 4, 5, 6, and 7 (Acceptance, Positive Refocusing, Refocus of Planning, Positive Reappraisal, Putting Into Perspective), the intervention indicator variable at the *α*=.05 level (2-tailed, and where relevant 1-tailed) was not statistically significant.

**Table 2:** Overview of outcomes following the SPARX intervention (n=22).

Primary outcomes: scale or subscale score	Desirable direction of improvement	Prescore (pooled groups A and B)	Postscore (pooled groups A and B)	Pre to post *t* test (pooled groups A and B)	Repeated measures ANOVA (pooled groups A and B)	Mixed models (multilevel regression models; comparing groups A and B)
CYRM^a^: Resilience	↑	47.389	44.389	Post ↓ (*P*=.02)	Intervention ↓ (*P*=.01)	Intervention ↓ (*P*=.02)
HSC^b^: Hopelessness	↓	4.458	3.667	Post ↓^c^ (*P*=.08)^d^	Intervention ↓ (*P*=.01)	Intervention ↓ (*P*=.02)^d^
CESDR^e^: Depression	↓	N/A^f^	N/A	Post ↑ (*P*=.02)	Intervention ↑ (*P*=.01)	Intervention ↑ (*P*=.08)
CERQ-Short^g^ subscale 1: Self-blame	↓	5.348	4.609	Post ↓ (*P*=.01)	Intervention ↓ (*P*=.02)	Intervention ↓ (*P*=.03)^d^
CERQ-Short subscale 2: Acceptance	↑	6.238	6.476	Post ↑ (*P*=.03)	Intervention ↓ (*P*=.07)	Intervention ↓ (*P*=.07)
CERQ-Short subscale 3: Focus on Thought/Rumination	↓	6.174	5.478	Post ↓ (*P*=.04)^d^	Intervention ↓ (*P*=.05)	Intervention ↓ (*P*=.04)^d^
CERQ-Short subscale 4: Positive Refocusing	↑	5.286	5.476	Post ↑ (*P*=.03)	Intervention ↓ (*P*=.08)	Intervention ↑ (*P*=.06)
CERQ-Short subscale 5: Refocus on Planning	↑	6.905	5.9524	Post ↓ (*P*=.02)	Intervention ↓ (*P*=.02)	Intervention ↓^c^ (*P*=.08, 2-tailed)^d^
CERQ-Short subscale 6: Positive Reappraisal	↑	7.714	6.905	Post ↓ (*P*=.02)	Intervention ↓ (*P*=.07)	Intervention ↓^c^ (*P*=.05, 2-tailed)^d^
CERQ-Short subscale 7: Putting Into Perspective	↑	6.381	5.762	Post ↓ (*P*=.04)	Intervention ↓ (*P*=.07)	Intervention ↓ (*P*=.01)
CERQ-Short subscale 8: Catastrophizing	↓	5.435	4.7826	Post ↓ (*P*=.05)^d^	Intervention ↓ (*P*=.04)^d^	Intervention ↓ (*P*=.03)^d^

^a^CYRM-12: Child and Youth Resilience Measure (12-item).

^b^HSC: Hopelessness Scale for Children.

^c^Approaches significance.

^d^Significant results (*P*<.05; 1-tailed, unless otherwise indicated).

^e^CESD-R: Centre for Epidemiologic Depression Scale-Revised.

^f^N/A: not applicable given insufficient data.

^g^CERQ-Short: Cognitive Emotion Regulation Questionnaire-Short.

### Hypothesis Two: Youth Who Completed the SPARX Program Were Expected to Experience an Increase in Factors Related to Resilience

Youth in the study did not show an increase in formal resilience indicators. The CYRM-12 intervention indicator was not statistically significant at the *α*=.05 level (2-tailed).

## Discussion

### Principal Findings

Overall, key findings suggest that youth who completed the SPARX trial learned new cognitive emotional regulation strategies to help support them in challenging maladaptive thought patterns. Youth also appeared to experience less hopelessness after engaging in SPARX. No formal indication of a decrease in depressive symptoms or an increase in resilience was noted after engaging in the SPARX program.

Youth who completed the SPARX program were expected to show a decrease in depressive symptoms, which was not evident in this pilot study. However, outcome measures suggest that, for Inuit youth in Nunavut, SPARX may be an effective program for decreasing feelings of hopelessness, as well as the cognitive emotion dysregulation signified by self-blame. Cognitive emotion regulation has consistently been linked to mental health, with those who possess more adaptive regulation strategies better able to cope with stressful or adverse life experiences [31]. Elevated self-blame, rumination, and catastrophizing are 3 factors known to be highly correlated with poor emotion regulation and mental health difficulties, such as depression and anxiety [32]. Youth who tend to ruminate on negative events, catastrophize, and self-blame are more likely to develop depressive symptoms. These youth have increased difficulty inhibiting and regulating their appraisal, which likely leads to more catastrophizing thoughts [33].

Although depressive symptoms on the whole did not decrease after youth trialed SPARX, results suggest a decrease in negative emotion regulation strategies. This is encouraging given the evidence that negative coping styles are associated with greater depressive symptoms [34]. With a reported decrease in self-blame, catastrophizing, and rumination, youth may develop enhanced coping strategies to deal with adverse life events. Hopelessness has been one of the most important mental health factors examined in attempts to understand suicidal behavior and has been linked to suicide in Indigenous populations [35,36]. It is encouraging that results show a decrease in hopelessness and an increase in cognitive emotion regulation strategies. This supports the need to further examine the effectiveness of SPARX on a larger scale as well as more longitudinally, as CBT therapies may be more effective over several months [37].

Youth who completed the SPARX program were expected to experience increased resilience after the intervention but outcome measures provided by youth did not suggest any change in the resilience measure. The small sample size may largely explain this finding. It is also possible that the resilience measure itself was not culturally relevant or appropriate. Despite the lack of significant changes recorded in the formal measures of resilience, there is reason to believe that a decrease in the hopelessness scale may promote and foster greater resilience [36]. It is possible that SPARX may prove to be an effective treatment for decreasing depressive symptoms and boosting resilience when assessed in a larger study.

Youth in Nunavut disproportionately struggle with mental health concerns in comparison to youth in other Canadian territories and provinces. Despite this disparity, Inuit youth lack access to mental health services, largely because of a shortage of staff to provide and assist with evidence-based treatments, a direct result of continued oppression and marginalization under enduring colonialism experienced by these communities [10]. Developing mental health programs that do not necessarily require staff support may be viable options in overcoming this barrier. Extant literature documents the success of computer-based CBT programs in increasing access to mental health resources for youth [38-40]. A review of over 100 studies evaluating the use of e-interventions for children and youth struggling with mental health needs showed that technology-assisted interventions are becoming an increasingly common form of service delivery in Australia, New Zealand, the United States, the United Kingdom, Canada, and the Scandinavian countries [41]. Thus, an increasing number of studies are overwhelmingly supportive of e-interventions, including serious games, as successful therapeutic tools. In addition to their clinical benefit, results suggest enhanced interest in and access to these services by youth, decreased costs compared with traditional services, and greater quality of life for those who access e-interventions [41]. Moreover, a growing field of research is specifically examining the use of these programs in remote and Indigenous communities, with promising results demonstrating the effectiveness of computer-based programs with these populations [38]. Digital interventions have demonstrated success in many remote and underserved communities, as they increase access, break down barriers, and fill key gaps in service provision [41-44].

This study aimed to establish whether SPARX would be an effective intervention for fostering resilience and decreasing the risk of depression with Inuit youth in Northern Canada. It appears as though SPARX may be a good first step for supporting youth with skill development for challenging maladaptive thoughts and providing behavioral management techniques such as deep breathing. Further larger-scale studies are required to understand whether SPARX or similar e-interventions can be effective in decreasing symptoms of depression.

### Limitations

We encountered several challenges during the implementation of this pilot study: (1) complications with youth recruitment, which resulted in the adoption of less rigid exclusion criteria; (2) challenges pertaining to the staffing of facilitators; (3) unavoidable deadline extensions due to staffing challenges, technological difficulties, and barriers in communication; and (4) a poorly matched control and intervention group when it came to sample size, duration, and frequency of play for youth in each group. The numerous challenges encountered caused this study to be drawn out over 9 months instead of the proposed 4.

As is common with pilot studies, the statistical power of our analysis was limited by a small sample size. Pilot studies are not expected to provide definitive statistical results, but rather to confer suggestive results for follow-up [45], and despite the lack of power we can report some statistically significant findings at the *α* level of .05. In addition, the “play now play later” procedures for this study were intended to control for the effects of fluctuations in mood due to daylight hours and fluctuating temperatures in the North. This was managed by having youth in the control group exposed to SPARX 2 months after youth in the intervention group completed their use. However, a strict deadline had to be set for the end of the study, and thus many youth in the control group were given a shorter time allowance for completing the SPARX program. Differences between the 2 groups in the frequency and duration of their SPARX play undermined the advantages of having a control group. Further, as a result of this deadline, there were fewer youth who participated in the control group. Finally, for ethical reasons, no participating youth were denied access to SPARX (ie, we ensured both group A and group B participants were all eventually given access to the intervention). Without a true control group where some participants did not receive SPARX, it is impossible to rule out confounds in the results. Unmatched sample sizes across the 2 groups, and an overall smaller-than-anticipated sample size, also weakened the scientific rigor of this study.

Another limitation was that the questionnaires used in this study, which were designed and systematized based on Western, Eurocentric concepts of depression, hopelessness, and resilience, did not properly access the experiences and expressions of mental health for Inuit youth in Canada. It is also possible that challenges with English language literacy confounded findings with Inuit youth participants despite staff being available to assist. It is possible that using local dialects (Inuinnaqtun and Inuktitut) for questionnaires would better support youth’s understanding of their mental health difficulties.

Finally, inconsistent access to the internet in most communities meant it was difficult to administer surveys; this also hindered data collection, participation monitoring, and recordkeeping. The high turnover of community facilitators created confusion with frequent changes in their roles and inadequate time provided for training, knowledge mobilization, and relationship development with youth participants. The conditions under which the trial was administered understandably created ambivalence among community facilitators, who were generally in favor of an intervention that might eventually benefit their communities but also struggled with the additional workload it imposed. All of these barriers, including short staff contracts, high staff turnover, youth attrition, inordinate mental health needs, lack of phone and internet access, and communication difficulties, are closely linked to continued colonization practices. Inuit youth in Canada continue to face systemic challenges and resource disparities as a result of poor government funding and a lack of consultation with communities for needs assessment and program development.

With these barriers and limitations in mind, we are also aware that this pilot study is rooted in a positivistic Western framework and was designed, conducted, and facilitated primarily by Qallunaat (non-Inuit). However, due to limited funding, this commissioned study had to be conducted remotely and included directives from the Government of Nunavut to involve local mental health workers, most of whom are not Inuk themselves, to administer the SPARX trial. This exploratory pilot served as a feasibility study fostering enthusiasm and cohesion among participating communities and scaffolding more culturally appropriate research. In a companion study [46], participating youth and community facilitators built much of the knowledge necessary through exit focus groups to facilitate such an endeavor. Indeed, efforts are now well underway to follow-up with a more community-directed and culturally meaningful study focused on articulating Inuit knowledge and research epistemologies through holistic, relational perspectives rooted in *Piliriqatigiinniq,* the Inuit concept for working collaboratively for the common good [20]. We recognize that research in Inuit communities must be collaborative and inclusive, underscoring “the right of colonized, Indigenous peoples to construct knowledge in accordance with the self-determined definitions of what they want to know and how they want to know it” [20](p12).

### Future Directions

We present here preliminary findings that suggest that serious games such as SPARX could lead to improvements in emotion regulation and, by association, depressive symptoms; however, conflicting findings suggest youth’s mood did not change after SPARX engagement. It will be important to replicate this study on a larger level, including more communities. It may also be beneficial to allow for online play. This will have the added benefit of increased rigor by allowing the research team to directly monitor engagement and frequency and length of play. It will be important to add a true control condition in the next trial to ensure that SPARX’s effectiveness can be more conclusively established. In addition, it will be important to gauge what mental health and mental illness mean to Inuit youth in Canada. This will help foster an understanding of more culturally appropriate questionnaires for accessing youth’s experiences with SPARX. Finally, using measures that have been validated with Inuit communities in Canada may help ensure that youth can more accurately report their mental health status. With a more culturally appropriate model for understanding Inuit youth mental health symptomology, an assessment of mental health changes attributable to the SPARX program may be more accurate.

Learning from the many barriers encountered here will allow for a more culturally competent and rigorous approach to conducting future research. It will be important to examine these barriers and to address them in any future protocol (such a follow-up study is currently underway). For example, having a community facilitator working specifically on SPARX will better support youth engagement, mitigate attrition, and foster greater trust between the youth and community facilitators. SPARX in schools might synergize well with institutional curricula and help with recruitment, engagement, and the generalizability of skills. Having a researcher on-site in the communities may help with supporting the community facilitator, troubleshooting any technological difficulties, and minimizing communication barriers. These extra supports, and the concomitant potential for more rigorous research with a larger sample size and true control group, will make an effective evaluation of the SPARX program more feasible. Furthermore, it may be advisable to hire a number of youth from each community to promote SPARX, advocate for the program, and help reduce the mental health stigma their communities may be facing.

Finally, it will be imperative to work with youth and communities to design, develop, and test an Inuit version of the SPARX program, tailored to fit the interests of Inuit youth and Elders in Canada and to increase engagement and effectiveness of the program. Such an initiative is underway. Existing studies suggest that incorporating one’s culture into an already compelling psychoeducational serious game has the potential to promote cultural esteem and community-based resilience, in addition to fostering individual resilience.

## Appendix

Multimedia Appendix 1 CONSORT extension checklist for pilot/feasibility trials.

## Abbreviations

CBT: cognitive behavioral therapy
CERQ-Short: Cognitive Emotion Regulation Questionnaire
CESD-R: Centre for Epidemiologic Depression Scale-Revised
CYRM-12: Child and Youth Resilience Measure-Short
GNAT: gloomy negative automatic thoughts
HSC: Hopelessness Scale for Children
