# Supplementary material for: Evaluating the Utility of a Psychoeducational Serious Game (SPARX) in Protecting Inuit Youth From Depression: Pilot Randomized Controlled Trial
Source: JMIR Serious Games. 2023 Mar 9;11:e38493. doi: 10.2196/38493 (PMC10037175; doi:10.2196/38493)
Supplement: Multimedia Appendix 1 [file games_v11i1e38493_app1.pdf]

# CONSORT 2010 checklist of information to include when reporting a pilot or feasibility randomized trial in a journal or conference abstract

| Item | Description | Reported on line number |
|---|---|---|
| Title | Identification of study as randomised pilot or feasibility trial | 3 |
| Authors * | Contact details for the corresponding author | |
| Trial design | Description of pilot trial design (eg, parallel, cluster) | 153 |
| Methods | | |
| Participants | Eligibility criteria for participants and the settings where the pilot trial was conducted | 110 |
| Interventions | Interventions intended for each group | 161 |
| Objective | Specific objectives of the pilot trial | 67, 84, 100 |
| Outcome | Prespecified assessment or measurement to address the pilot trial objectives** | 206, 215, 222, 233 |
| Randomization | How participants were allocated to interventions | 161 |
| Blinding (masking) | Whether or not participants, care givers, and those assessing the outcomes were blinded to group assignment | N/A |
| Results | | |
| Numbers randomized | Number of participants screened and randomised to each group for the pilot trial objectives** | 289 |
| Recruitment | Trial status† | |
| Numbers analysed | Number of participants analysed in each group for the pilot objectives** | 289 |
| Outcome | Results for the pilot objectives, including any expressions of uncertainty** | 289, 322 |
| Harms | Important adverse events or side effects | N/A |
| Conclusions | General interpretation of the results of pilot trial and their implications for the future definitive trial | 461 |
| Trial registration | Registration number for pilot trial and name of trial register | NCT05702086 clinicaltrials.gov |
| Funding | Source of funding for pilot trial | 91 |

Citation: Eldridge SM, Chan CL, Campbell MJ, Bond CM, Hopewell S, Thabane L, et al. CONSORT 2010 statement: extension to randomised pilot and feasibility trials. BMJ. 2016;355.

**this item is specific to conference abstracts*

***Space permitting, list all pilot trial objectives and give the results for each. Otherwise, report those that are a priori agreed as the most important to the decision to proceed with the future definitive RCT.*

*†For conference abstracts.*
